# Radiotherapy of the Primary Disease for Synchronous Metastatic Cancer: A Systematic Review

**DOI:** 10.3390/cancers14235929

**Published:** 2022-11-30

**Authors:** Youssef Ghannam, Adrien Laville, Youlia Kirova, Igor Latorzeff, Antonin Levy, Yuedan Zhou, Vincent Bourbonne

**Affiliations:** 1 Radiation Oncology Department, Centre Paul Papin, Institut de Cancérologie de l’Ouest, 49055 Angers, France; 2 Radiation Oncology Department, CHU Amiens-Picardie, 80000 Amiens, France; 3 Radiation Oncology Department, Institut Curie Paris, CEDEX 05, 75248 Paris, France; 4 Radiation Oncology Department, Bât Atrium Clinique Pasteur, 31300 Toulouse, France; 5 Radiation Oncology Department, Gustave Roussy, Université Paris-Saclay, 94805 Villejuif, France; 6 Faculté de Médecine, Université Paris-Saclay, 94270 Le Kremlin-Bicêtre, France; 7 Radiation Oncology Department, University Hospital, 29200 Brest, France

**Keywords:** primary tumor, locoregional treatment, metastatic cancer, oligometastatic cancer, radiotherapy

## Abstract

**Simple Summary:**

Local radiation treatment of the main tumors in patients with synchronous metastatic illness has traditionally only been used for palliative purposes. The management of patients with de novo metastatic cancer is undergoing a revolution with the advent of new systemic therapies enabling longer overall survival with enhanced quality of life. Numerous studies have looked into the potential survival advantage of treating localized primary tumors at the oligometastatic or oligopersistent stage.

**Abstract:**

In the case of synchronous metastatic disease, the local treatment of primary tumors by radiotherapy has long been reserved for palliative indications. The emergence of the concept of oligometastatic and oligopersistent diseases, the advent of new systemic therapies enabling longer overall survival with an enhanced quality of life, a better understanding of the biologic history of metastatic spread, and technical advances in radiation therapy are revolutionizing the management of patients with de novo metastatic cancer. The prognosis of these patients has been markedly improved and many studies have investigated the survival benefits from the local treatment of various primary tumors in cases of advanced disease at the time of diagnosis or in the case of oligopersistence. This article provides an update on the place of irradiation of the primary tumor in cancer with synchronous metastases, and discusses its interest through published or ongoing trials.

## 1. Introduction

While systemic treatments (chemotherapy, targeted therapies, hormonal therapies, immunotherapies, etc.) are the standard-of-care of synchronous metastatic cancers, local treatment of the primary tumors by surgery or radiotherapy (RT) was mainly used as palliative or symptomatic management (pain, bleeding, etc.). The progress of systemic treatments in recent years has changed the prognosis of these patients, with significantly prolonged survival [1] and sometimes achieved complete remission for several years. This raised the question of treatment of the primary tumors. For some primary diseases, locoregional therapy (LRT) for the intact primary tumor has been hypothesized to improve overall survival (OS), but retrospective series and clinical trials have reported conflicting results. Pooling the data from 4952 patients with various histology subtypes, of whom 1558 received RT and 912 surgery, Ryckman et al. did not find a benefit in progression-free survival (PFS) nor overall survival (OS) [2]. Local treatment of the primary was associated in an OS benefit but only in low metastatic burden patients (HR 0.67, 95% CI 0.52–0.85) while surgery did not improve OS whatever the metastatic burden. When sub-analyzing the results, differential responses may appear depending on the primary and histology. This article thus provides an update on the role of RT on the primary tumors in breast, prostate, and lung cancers with synchronous oligometastatic or oligopersistent disease, and discusses its value through published or ongoing trials.

### 1.1. Rational

#### 1.1.1. Biological Rational

Stephen Paget formulated the “seed and soil” theory in 1889, whereby metastatic spread is not a random process, but is governed by cooperation between the tumor cells “seeds” and the host organ “soil” [3]. An upstream preparation for metastatic spread requires a suitable microenvironment in the distant organ. A pre-metastatic niche is necessary for metastatic development [4]. This microenvironment consists of a set of immune cells and extracellular matrix proteins forming the metastatic bed. The primary tumor initiates the process of niche formation in distant organs not only by producing growth factors that increase the proliferation of stromal cells, but also by recruiting bone marrow-derived hematopoietic cells to the premetastatic niche [5]. In addition, myeloid precursors are recruited by the primary tumor via cytokines to allow tumor cells to remain undetected by the immune system and thus allowing metastatic development [6]. Primary tumors also secrete exosomes, nanovesicles of 40 to 100 nm in diameter involved in intercellular communication, allowing the exchange of proteins and nucleic acids in particular [7,8].

By secreting a large number of exosomes, primary cancer cells not only influence proximal tumor cells and stromal cells in the local microenvironment, but also have distant systemic effects. They modulate the immune system by stimulating the induction of apoptosis of cytotoxic T cells or the inhibition of natural killer lymphocyte cytotoxicity. These vesicles can also stimulate angiogenesis by interaction with endothelial cells when secreted under hypoxic conditions [9,10]. There is a real molecular communication between the primary tumor and the metastases.

In addition, the primary site may be the source of circulating tumor cells (seeding) which may themselves recolonize the primary tumor (self-seeding) [11,12]. Thus, local irradiation of the primary tumor could suppress this signaling that favors metastatic development. Moreover, lymphocyte activation via DAMPS (damage-associated molecular pattern), a set of pro-inflammatory molecules derived from radiation-induced cell death, could induce an antitumor immune response [13].

A better understanding of the molecular interactions is needed to adapt the therapeutic choices according to the biological profile in order to have a treatment benefit without inducing more toxicity.

#### 1.1.2. Synchronous Metastatic Cancers

The survival of patients with de novo metastatic cancer is very heterogeneous, probably due to the fact that there are several distinct groups of metastatic cancers. Hellman and Weichselbaum named one of the groups: “oligometastatic cancers”. It is an intermediate and indolent disease stage with a limited number of metastatic sites (classically fewer than three to five), and is characterized by slow tumor growth (Hellman). Eradicating the metastatic lesions could improve patients’ survival [14].

However, a formal demonstration of the benefit of treatment of oligometastases is still lacking. The SABR COMET trial compared stereotactic irradiation of the metastatic sites in addition to systemic treatment with systemic treatment alone in 99 patients with oligo recurrent or metastatic (after initial treatment of primary tumors) [15].

The primary tumor sites included lung (*n* = 18), breast (*n* = 18), colon (*n* = 18), prostate (*n* = 16), and other localizations (*n* = 29). Eight-year OS was 27.2% in the SABR arm vs. 13.6% in the control arm (hazard ratio (HR): 0.50; 95% confidence interval (CI): 0.30–0.84; *p* = 0.008). The heterogeneity of the population makes it impossible to conclude on the value of irradiation of metastatic sites, especially in breast cancer. However, for de novo oligometastatic cancer, the idea of combining maximalist systemic treatments with ablative treatment of the metastases and local treatment of the primary could be an interesting strategy.

## 2. Irradiation of the Primary Disease for Synchronous Metastatic Breast Cancer

### 2.1. Retrospective Series

Retrospective studies performed on local treatment of primary tumors examined local treatment options combining surgery with or without postoperative radiotherapy. These studies were mostly performed in a single-center, and presented a variety of methodologies with contradictory findings.

For palliative treatment, local irradiation of the primary tumor seems to control the symptomatology with an acceptable morbidity. In an analysis of the Surveillance, Epidemiology, and End Results (SEER) database of 3660 patients with stage T4M1 breast cancer, 1558 (43%) received surgery (15%), radiation (15%), or both (9%). Symptom improvements were observed in almost 50% of patients, but with an increase in local morbidity (mainly lymphoedema after axillary surgery and neuropathic pain) in 20% of patients who were initially asymptomatic [16].

#### 2.1.1. Impact of Local Treatment on Survival

The first retrospective study to show the benefit of local treatment on the primary tumor was conducted between 1990 and 1993, including 16,023 patients (4.1%) with metastatic breast cancer at the outset. Breast surgery was performed in 9162 patients (57.2%), of which 61.7% were mastectomies. Radiation therapy was performed in 5806 patients, most of whom had undergone surgery [17]. However, the radiation targets (the breast or metastatic lesions) were not specified. The 3-year OS was 17.3% in the no-surgery group, 27.7% in the partial mastectomy group, and 31.8% in the total mastectomy group. 

Since then, numerous retrospective studies have shown the benefit of local treatment by surgery with or without complementary irradiation on survival or radical radiotherapy, fueling a debate that is still ongoing (Table 1).

In a more recent retrospective study published by Stahl et al. in 2021, a survival benefit was observed for patients who received either systemic therapy and surgery (HR 0.723; 95% CI 0.671–0.779) or systemic therapy, surgery, and radiation (trimodality: HR 0.640; 95% CI 0.591–0.694) (both *p* < 0.0001) compared with systemic therapy alone [37]. However, once again the LRT seems undistinguishable from distant RT to metastatic sites. Surprisingly, oligometastatic diseases represented 38% of the patients in this series, which is much higher than the usual series, even in academic centers. Furthermore, systemic therapy was used in a small proportion of patients (40% in 2014–2015) which is questionable in stage IV patients. 

In addition, the response to systemic treatment seems to be important to consider since some metastatic patients who have effective treatment and stable disease have a better survival than patients with a locally advanced disease without response to systemic treatment [38]. 

In several retrospective studies, patients with isolated bone metastases appeared to benefit the most from local therapy in terms of overall survival [17,18,39]. Some major prognostic factors of overall survival in favor of local therapy were frequently reported: R0 surgical resection, young age of the operated patients (50–60 years), oligometastatic involvement (one metastasis versus several metastases). Other criteria have been identified: tumor size, hormone receptor status and axillary lymph node involvement. Patients with cancer expressing hormone receptors or HER-2 amplification (*p* = 0.004) would benefit more from local treatment, probably due to the effectiveness of systemic treatment [28].

#### 2.1.2. Impact of Exclusive Irradiation on Survival

Two retrospective French series of studies have examined the impact of exclusive radiotherapy as a local treatment for the primary tumor. In the Curie-Huguenin study reported by Le Scodan et al. of 18,753 patients with breast cancer treated between 1980 and 2004, 598 (3.2%) had metastases at diagnosis [33]. Of the 581 eligible patients, 320 received local treatment, by exclusive radiation in 249 patients (78%), by surgery followed by radiation therapy in 41 patients (13%), or by surgery alone in 30 patients (9%). The average radiation dose was 48 Gy in the breast, with the possibility of a local boost of 22 Gy. With a median follow-up time of 39 months, the probability of survival at 3 years was 43.4% versus 26.7% for the groups with and without local treatment, respectively (*p* = 0.00002).

In the multifactorial analysis, radiotherapy was an independent factor that significantly improved overall survival. The improvement in survival was particularly marked in women with visceral metastases. Authors concluded that radiation therapy could be proposed as an alternative treatment to surgery in patients with metastatic cancer at the time of diagnosis.

The second study by the Gustave Roussy [34] was conducted between 1990 and 2003, among 9138 patients; 308 patients had stage IV disease. The majority of patients (2/3) had a single metastatic site and 49% had non-visceral metastases at diagnosis. LRT was performed in 80% of patients (*n* = 239) either by exclusive radiation (*n* = 147) or by breast and axillary surgery with or without postoperative radiotherapy (*n* = 92). In the operated group, the cancers were of smaller sizes, lower in tumor grade, had less clinical axillary lymph node involvement, and had a lower tumor burden than in the exclusive radiation group. With a median follow-up of 6.5 years, locoregional control was achieved in 85% of patients. The probabilities of metastasis-free survival and overall survival at 3 years were 20% and 39% with exclusive radiation therapy and 39% and 57% with surgery, without significant difference.

Several meta-analyses of these studies, including Gera’s work published in 2020, supported LRT to improve survival in these patients with de novo metastatic cancer [40,41].

These results, often from uni- or multifactorial analyses, are sometimes contradictory and should be interpreted with caution because of potential selection bias. The survival advantage of patients undergoing surgery could be explained by selection bias [23]. Published analyses indicate that there is an imbalance between the groups and those patients with lower tumor burden, less dissemination and with a better physiology state (age, comorbidities) are more likely to be candidates for LRT. For example, in the study published by Blanchard et al., it was found that at least 25% of cancers that were operated and 3% of the unoperated tumors were reclassified [25]. This would suggest that their initial presentation was stage I, II or III and only after completion of the extension work-up, they were reclassified as stage IV. This means that for some patients the indication for surgery was based on curative intent and not for palliative purposes [42]. The only way to overcome these selection biases is through prospective randomized trials.

### 2.2. Prospective Studies

Published prospective studies and ongoing trials on this topic follow two distinct designs (Table 2). The first one was where the patients enrolled received systemic therapy before any LRT. Then, they were registered and if they did not progress after chemotherapy, they were randomized in the LRT group (followed by systemic therapy) or continued systemic therapy alone. The Indian Tata Memorial Center trial included 350 patients under 65 years, with de novo metastatic breast cancer between 2005 and 2013 [43]. Patients were randomized to LRT or no LRT. Among the 350 patients, 336 had unresectable tumors. These received a neoadjuvant chemotherapy and were randomized according to response to receive local or no treatment. One hundred and seventy-three patients underwent surgery (72% mastectomy), and 80% received adjuvant radiotherapy. This trial did not show any benefit in terms of 2-year OS: 41.9% (95% CI 33.9–49.7) in the LRT group versus 43% (35.2–50.8) in the no LRT group. In the multifactorial analysis, global survival was independently associated with hormone receptor expression and a low number of metastatic sites at initial presentation. The site of metastasis at initial presentation was not significantly associated with overall survival. Overall survival in both groups was lower than reported in Western countries, possibly due to the delay in diagnosis. In addition, 107 patients (31%) had HER2-expressing cancers, but due to financial constraints, 98 patients in this subgroup (92%) did not receive anti-HER2 targeted therapy. In this study, LRT resulted in significantly longer locoregional progression-free survival compared with the no-treatment group. This was tempered by the authors, who suggest that initial LRT cannot be justified for local symptom control alone, because only a minority (10%) of patients in the no-local-treatment group still underwent surgery for local palliative reasons.

The recently published North American trial enrolled 390 participants, 256 were randomly assigned: 131 to continued systemic therapy and 125 to early LRT (surgery and radiotherapy) [45]. The 3-year OS was 67.9% without and 68.4% with early LRT (hazard ratio = 1.11; 90% CI, 0.82 to 1.52; *p* = 0.57). Locoregional progression was less frequent in the LRT group (3-year rate: 16.3% *v* 39.8%; *p* < 0.001). No difference in quality of life was observed between the two arms. Overall survival by tumor subtype for the 20 women with triple-negative breast cancer tended to be worse with the addition of LRT (HR = 3.50).

The Japan Clinical Oncology Group (JCOG) 1017 PRIM-BC is an ongoing trial that was conducted to confirm the superiority, in terms of overall survival, of local treatment of the primary disease with surgery [46]. All patients received a standard systemic treatment after the first registration. After 3 months, patients with non-progressive disease were randomized to surgery with systemic therapy or to systemic therapy alone. The study protocol did not specify whether patients in the surgery group received postoperative radiotherapy.

The second design of the prospective trials was where patients were directly randomized to either to systemic therapy alone or to LRT followed by systemic therapy.

The Turkish randomized trial MF07-01 included 274 patients between 2007 and 2012 [44]. They received local treatment (surgery and radiotherapy in case of conservative surgery) followed by systemic treatment (for 138 patients) or systemic treatment alone (for 136 patients). There was no stratification on baseline characteristics, which explains some of the imbalances between the groups (hormone receptor expression, 85.5% vs. 71.8%, and triple-negative tumors, 7.3% vs. 17.4% in the groups with and without local treatment, respectively). It should be noted that there was a relatively high number of single metastases (30%), while the extension workup included (18 F)-fluorodeoxyglucose PET. Overall survival was significantly prolonged by LRT (at 5 years 41.6% vs. 24.4% *p* = 0.005). An unplanned subgroup analysis showed a significant overall survival advantage with LRT in patients with hormone receptor-positive but non-HER2 cancers, patients with exclusive bone metastases, and patients younger than 55 years. The benefit of local treatment appears particularly clear in the case of single bone metastases.

The Austrian prospective randomized phase III ABCSG-28 POSYTIVE trial attempted to assess median survival by comparing primary surgery followed by systemic therapy to systemic therapy alone in de novo stage IV breast cancer [47]. This trial did not reach full accrual. Ninety-three patients were included against 254 subjects needed. This trial could not demonstrate an overall survival benefit in favor of surgery. Patients randomized to systemic therapy had a median survival rate of 54 months, compared with 34 months in the surgical group. Although the trial was not sufficiently powered, the authors said this trend indicates that caution should be exercised regarding primary surgery in the setting.

Despite several studies on the subject, there is, to date, no clear recommendation or consensus for radiotherapy of the primary disease in synchronous metastatic breast cancer. The negative results of three prospective trials encourage caution regarding LRT, which have to be systematically discussed in multidisciplinary concertation meetings. However, it would appear that LRT by surgery followed by radiation after response to initial systemic therapy would be a good option, particularly in young patients with hormone receptor-expressing, non-HER2, oligometastatic cancer that tends to have bone-only metastases. The systemic treatment remains the standard first-line treatment in the case of metastatic disease and it appears that LRT should not interfere with its implementation, hence the interest in knowing how the new targeted molecules are associated with radiotherapy [48,49,50].

## 3. Irradiation of the Primary Tumor for Metastatic Prostate Cancer

Despite implementation of individualized screening, about 10% of patients are diagnosed with initially metastatic prostate cancer [39]. For a long time, metastatic prostate cancers were univocally considered to have a poor prognosis and only systemic treatments were indicated. Many retrospective studies were published suggesting a benefit from local radiotherapy for these patients. Most of them are large population-based studies using propensity-scores [51,52,53,54,55,56,57,58] (Table 3). Two clinical trials and one meta-analysis have suggested the value of local prostate radiotherapy in this context to improve clinical outcomes.

### 3.1. HORRAD Trial

HORRAD was the first prospective randomized trial published. This study included 432 metastatic prostate cancer patients, randomized between hormone therapy alone or combined with prostate radiotherapy [59]. The primary endpoint was overall survival, and the secondary endpoint was time to biochemical progression. The median age of the population was 68 years old. The median PSA level was 145 ng/mL. The dose prescribed was 70 Gy in 35 fractions of 2 Gy or 57.76 Gy in 19 fractions of 3.04 Gy. The GTV included the prostate and extensions, the base of the seminal vesicles. Regarding the planning target volume, 1 cm margin in conventional radiotherapy was applied or 8 mm if a position verification protocol with fiducial marker was implanted. Of the patients, 67% had more than five bone metastases at the time of randomization (high volume metastatic burden). With 47 months median follow-up, no difference in overall survival was demonstrated (45 vs. 43 months, HR 0.90; 95% CI [0.70–1.14]. The non-significance could be explained by a lack of statistical power. In the subgroup analysis, patients with fewer than five metastases (*n* = 160) had a trend towards better overall survival (HR 0.68; 95% CI 0.42–1.10 *p* = 0.063). Local radiotherapy was associated with 3-month improvement in time without PSA increase (HR = 0.78; 95% CI: [0.63–0.97]; *p* = 0.02). As suggested by the absence of clear OS benefit despite a benefit in biochemical response, PSA level only should not be used as a surrogate for OS. 

A supplementary analysis by Boevé et al. assessed side effects and quality-of-life in this cohort [60]. Apart from local symptoms, there was no significant difference in mean scores on the quality of life items evaluated by QLQ-C30 et QLQ-PR25, with a difference of 10 points from the baseline considered relevant. More frequent urinary and bowel symptoms and diarrhea were found in patients in the prostate radiotherapy group within 3 months after treatment. The bowel symptom score was significantly higher in 22% patients treated with radiotherapy at two years follow-up (HR = 8; CI 95% [4.8–11.1]).

### 3.2. Stampede Trial

STAMPEDE was a randomized controlled trial who evaluated the benefit of prostate radiotherapy in addition to androgen deprivation therapy in patients with hormone-sensitive metastatic prostate cancer [61]. Totally, 2061 patients were enrolled in this two arms phase III trial randomizing the combination of prostate radiation therapy with androgen suppression or androgen suppression alone. Selected patients were newly diagnosed with metastatic prostate cancer, without prior radical treatment and with metastatic disease confirmed by standard imaging. Radiation could be delivered at a dose of 55 Gy in 20 daily fractions of 2.75 Gy or 36 Gy in 6 weekly fractions of 6 Gy. The planned target volume included the entire prostate +/- seminal vesicles. The primary endpoint was overall survival and failure-free survival (FFS). Secondary endpoints were local symptomatology, progression-free survival (PFS) and metastatic progression free survival. Biological relapse was defined as an increase of at least 50% in PSA level. Patients were divided into subgroups according to their initial metastatic burden based on imaging data (CT, MRI, and scintigraphy). High metastatic burden was defined according to CHAARTED criteria (≥ four bone metastases with ≥ one outside the pelvis and vertebrae, or visceral metastases, or both). Patients who did not meet these criteria were classified as low burden. 89% of patients had initially bone metastases. The median PSA level before androgen suppression was 98 ng/mL and 97 ng/mL respectively in each arm. The population median age was 68 years old. Gleason score was ≥ 8 in 79% of cases. 40% and 54% of patients had low and high metastatic burden, respectively. It was unknown for 6% of them. In the entire cohort, no significant benefit was found in overall survival with local radiotherapy (HR 0.92; 95% CI [0.8–1.06]; *p* = 0.27) with a median follow-up of 37 months. Failure-free survival was significantly improved in the radiotherapy arm (HZ= 0.76; 95% CI 0.68–0.84; *p* = 3.4 × 10^−7^). Patients with a low metastatic burden (*n* = 819) had significantly better overall survival and failure-free survival (HZ = 0.68; 0.52–0.90; *p* = 0.007). The addition of radiotherapy show a 8% improvement in overall survival in this previously planned subgroup analysis (73% vs. 81% (HR: 0.68; CI 95%: [0.52–0.90]; *p* = 0.007). The interaction test was significant (*p* = 0.0098). The hypo fractionated 55 Gy in 20 fractions regimen appeared to be more effective in terms of failure-free survival (HR 0.69, 95% CI 0.59–0.80; *p* < 0.0001). There was 65% of urinary and 47% of digestive toxicity compared with 71% and 62% in favor of the weekly arm. Prostate radiotherapy appeared to be well tolerated with 4% grade 3–4 toxicity compared to 1% with androgen deprivation alone. For the 533 patients for whom data were available, 15% of patients in the control arm and 13% in the radiotherapy arm had grade ≥ three adverse events found at two years of follow-up.

Ali et al. investigated the effect of prostate radiotherapy according to the severity of metastatic spread in the STAMPEDE cohort [39]. More than 2000 patients were randomized, with less than 2% of patients with four or more bone metastases in the spine alone. The survival benefit decreased while increasing number of metastases up to a threshold of three bone metastases. A gain in overall survival was correlated with the number of bone metastases: 8.5%, 6.2% and 5.8% at 3 years follow-up in patients with one, two and three bone metastases respectively. No survival benefit was found in patients with visceral metastases or with strictly more than three bone metastases. For relapse-free survival, nine bone metastases were found as a threshold for benefit. The interaction between the number of bone metastases and treatment adjusted for age, PSA level before androgen suppression, T stage, Gleason score, N stage, metastatic sites, docetaxel use, and RT schedule showed similar results for OS and PFS. In the subgroup analysis, for patients with three or fewer bone metastases with or without non regional lymph node and no visceral metastases, local radiotherapy improved overall survival (3-year survival 85% vs. 75%, HR = 0.64 IC 95% [0.46–0.89]). No survival benefit was associated with four or more bone metastases with or without non-regional lymph node involvement. Classifying low metastatic burden patients as three or fewer bone metastases, regardless of location, with or without non-regional lymph node involvement, with no visceral metastases, the results were significant in overall survival (HR = 0.62 CI 95% [0.46–0.83] *p* = 0.01) and failure-free survival (HR = 0.57 CI 95% [0.47–0.70] *p* = 0.001. The effect of radiotherapy on OS and FFS within patients with low-burden disease did not rely on age, pre-ADT PSA level, World Health Organization performance status, Gleason score, tumor stage, regional nodal stage and schedule. This study supports the value of local radiotherapy in patients with a low number of bone metastases evaluated by conventional imaging.

### 3.3. STOP-CAP Meta-Analysis

The STOP-CAP meta-analysis pooled the two randomized trials HORRAD and STAMPEDE (*n* = 2126) [62]. No significative improvement in overall survival (HR 0.92, 95% CI 95% [0.81–1.04], *p* = 0.195) or progression-free survival (HR 0.94, CI 95% [0.84–1.05], *p* = 0.238) was found. Biologic progression-free survival (HR 0.74, CI 95% [0.67–0.82], *p* = 0.94 × 10^−8^) and failure-free survival (HR 0.76 CI 95% [0.69–0.84] *p*= 0.64 × 10^−7^) were improved. The interaction between the number of metastases (<5 vs. >5) and survival was significant (HR = 1.47 CI 95% [1.11–1.94], *p* = 0.007).

Although many patients classified as having a low metastatic burden, as defined by the HORRAD study, are also classified as having a low metastatic burden as defined by the CHAARTED criteria, the definition of tumor volume level remains heterogeneous between these two studies. Modern imaging techniques and molecular signatures would improve the accuracy of patient selection. A number of patients classified as having low metastatic burden would be likely classified as high burden using Choline-PET or PSMA-PET [63]. The benefit of local treatment according to the number of spinal metastases could not be addressed by the analysis of Ali et al. because only 2% of the STAMPEDE cohort had exclusive spinal bone metastatic involvement [39].

### 3.4. Ongoing Trials

Among the ongoing studies, the PEACE 1 trial is a four-arm multicenter study comparing the combination of androgen suppression, docetaxel chemotherapy +/- prostate radiotherapy (74 Gy in 37 fractions) +/- abiraterone acetate and prednisone. Results regarding the value of local radiotherapy are pending [64]. The NCT03678025 study conducted by the SWOG will evaluate the combination of systemic treatment with local treatment (surgery vs. prostate radiotherapy. Other studies are being conducted to answer the question of a combination of radiotherapy on the primary and oligometastases. PRESTO (prostate-cancer treatment using stereotactic radiotherapy for oligometastases ablation in hormone-sensitive patients) is an ongoing two-arm, multicenter phase III randomized trial. The objective is to evaluate the efficacy of stereotactic radiotherapy applied to all oligometastases in patients with hormone-sensitive oligometastatic prostate cancer, Table 4 [65,66,67,68,69,70,71,72,73].

Local control of the primary matters in selected newly diagnosed hormone-sensitive metastatic prostate cancer. For the majority of patients, prostate radiotherapy could provide a survival benefit with transient and manageable side effects. Although radiotherapy is well tolerated, patients should be informed that radiation is associated with more urinary symptoms and potentially chronic diarrhea. Selection criteria are not consensual and many other questions remain: radiation schedule, technical modalities, association with metastases-directed therapy. The latest recently published international and national guidelines recommend radiation to the primary [74,75]. An ongoing investigation of predictive molecular signatures and advances in nuclear imaging with the use of standardized indices to assess metastatic burden could help with better stratification.

## 4. Treatment of the Primitive Site in Metastatic Lung Cancer Patients

Lung cancer remains the leading cause of death worldwide with a high percentage being diagnosed as stage IV disease [76]. The arrival of immune checkpoint inhibitors (ICI) in lung cancer patients has completely modified the treatment of those patients, and especially patients with non-small cell lung cancer (NSCLC). At first-line [77,78] and second-line [79,80] treatments, both progression-free survival (PFS) and overall survival (OS) were significantly improved, especially when selecting patients based on the level of PD-L1 expression. Specific biomarkers such as EGFR mutations or ALK rearrangements were identified and could be directly targeted, with prolonged survival when compared to the usual chemotherapy-based regimen [81,82,83,84,85]. Clinicians face new entities of patients, such as long-responders to ICIs or oligometastatic/oligopersistent patients [86], in whom a curative objective could possibly be considered.

With this intent, radiotherapy could be delivered to lower the tumor burden [4,11,12] and possibly increase the PFS/OS. Abscopal responses were also described, yet poorly understood. Several technological advances have been made since the 2000s. Stereotactic radiotherapy allows the delivery of a high dose per fraction in 3–8 fractions, with a high tumor conformation resulting in high local control and a low risk of toxicity [87].

Local treatment has several potential advantages: prevention or treatment of eventual symptoms, prevention of primary/secondary seeding and maintenance with the same treatment and thus differing treatment changes [88].

To this day, several trials have focused on the impact of radiotherapy in metastatic lung cancer. Given the clear differences between NSCLC and small-cell lung cancer (SCLC) but also the lack of data in SCLC patients, this article only focuses on NSCLC patients. Of note, the benefits of a lower dose as thoracic consolidation were assessed in a randomized trial focusing on SCLC patients. The OS benefit was most pronounced when only patients with residual thoracic disease were included. To our knowledge, the CREST trial [89] remains the single published RCT in SCLC patients. Regarding NSCLC patients, local radiotherapy to the primary was investigated more deeply, but with very heterogenous populations.

### 4.1. Palliative Radiotherapy

According to the NCCN guidelines, local radiotherapy is recommended for palliation or prevention of symptoms such as pain, bleeding or obstruction [90]. In a cohort of 78 patients, palliative thoracic radiotherapy was associated with pain relief in 85.9% in the patients [91]. An improvement of the performance status (PS) was also reported [92], palliative radiotherapy being the possible bridge between palliative care only and systemic treatments [93].

### 4.2. Oligometastatic NSCLC

The oligometastatic NSCLC stage has been defined with a maximum of five metastases among three or fewer organs, as assessed with ^18^F-FDG positron emission tomography and brain imaging [94]. Mediastinal lymph nodes are not considered as metastases.

Local radiotherapy to metastatic sites, among which (but not limited to) lung metastases, achieves prolonged survival in selected patients [14,95,96,97,98]. These interesting results were first described on retrospective cohorts but later confirmed in several phase II trials. The main concern for patients under systemic treatment is the development of acquired resistance. Locally directed treatment such as radiotherapy could thus increase the PFS and possibly the OS in selected patients with indolent diseases [99]. The benefit of local therapy seems irrespective of the mutational status. Data should, however, be analyzed separately given the different PFS and OS between patients with and without targetable mutations.

#### 4.2.1. NSCLC without Targetable Mutations

One of the largest retrospective cohorts was based on the analysis of 186 patients with oligometastatic NSCLC that were either treated with surgery, local radiotherapy to the primitive (9%), to metastases (17%) and 20% to the primitive and the metastases; the rest did not receive radiotherapy. Radiotherapy was associated with a longer overall survival benefit (*p* = 0.04) but only after propensity score matching [100]. Published meta-analyses are limited by the small number of patients treated with high-dose radiotherapy, as well as a high risk of selection bias. For instance, a meta-analysis reported a 52% decrease in the 1-year death rate when delivering local treatment (74.9% in patients with local treatment and 32.3% in locally untreated patients). The number of metastases was the main prognostic factor [101]. In a meta-analysis aggregating the results of 21 (mainly) retrospective studies, the overall survival reached 20.4 months, with a 1-year survival probability of 70% [102].

Focusing on patients treated with ICIs, robust data remain limited. In a cohort of 148 patients with 38 oligoprogressive patients, switching the therapy group was not superior to continuation of the same ICI with added RT to the progressive lesions [103].

Prospective trials focusing on the benefit of local radiotherapy are very heterogenous regarding their design and treatment modalities. While several phase III trials are ongoing, only phase II results are available. Pre-treatment PET-CT was mandatory in only four out of six trials. The definition of the oligometastatic state varied between fewer than five and fewer than six sites. Among the 209 included patients, 170 patients (81.3%) received either surgery or radiotherapy (normofractionated, moderately hypofractionated or stereotactic) to the primitive and synchronous lesions [14,95,96,97,104]. In trials in which the overall survival for NSCLC-patients was available, OS ranged from 13.5 to 41.6 months, whereas median PFS ranged from 11.2 to 23.5 months.

For instance, focusing on 49 patients evaluated with a PET-CT (≤ three metastatic sites), the study by Gomez et al. constituted the largest prospective cohort dedicated to oligometastatic NSCLC. OS increased from 17 to 41.2 months (*p* = 0.02) [14,104].

Similar results were found but on smaller or non-NSCLC exclusive cohorts. Bauml et al. included 45 NSCLC patients in which 67% were treated with SBRT and pembrolizumab. In this single arm phase II study, the median OS reached 41.6 months [105]. Of note, patients were included only after the completion of SBRT. With 99 included patients but only 18 patients with NSCLC (18.2%), Palma et al. were able to validate the benefit of local radiotherapy among a variety of oligometastatic cancers, with a median OS of 50 months (vs. 28 in the control arm) [106]. To our knowledge, Palma et al. and Iyengar et al. [107] conducted the two single published prospective studies in which SBRT was mandatory. The main differences between the two were the cancer selection with only NSCLC patients in the Iyengar et al. study and the clinical setting. In the SABR-COMET trial, SBRT was delivered in case of oligorecurrence whereas in the study by Iyengar et al., only synchronous stage IV NSCLC were included.

As presented by Levy et al. [94] and actualized for this review in Table 5, RT modality varied greatly among these prospective trials. Even when SBRT was mandatory, prescriptions differed significantly from one study to the other. Similarly, the rates of patients with brain metastases varied greatly from one study to another.

Of note, patients with EGFR mutations could be included in some trials, with the rates reaching 12–20% [14,104] or even 43.2% [98]. In contrast, in a study in which patients with oncodrivers were excluded, local therapy increased the median PFS by only 6.2 months [107]. A separate focus on mutated-NSCLC seems necessary.

#### 4.2.2. NSCLC with Targetable Mutations: EGFR, ALK, ROS1

Interesting results were also obtained in patients with EGFR mutations [109]. Approximately half of recurrences after EGFR-targeted therapy occur first in the primary or pre-existing metastatic sites [88]. The primary lung tumor size appears as the strongest risk factor for failure in the original sites. In some reports, the local recurrence rate even reaches 60% as the site of first failure and the only site of failure for 30% [110]. Given the indolent pattern of certain NSCLCs under tyrosine-kinase inhibitors (TKIs), the evidence for benefit from RT seems more robust, with the lack of phase III trials.

Radiotherapy was evaluated as a consolidation treatment in 145 patients under TKIs, with 35.2% having received radiotherapy on the primitive and the metastases, 37.9% on either the primitive or the metastases and 26.9% having received no radiotherapy. Median PFS and OS of 20.6 months and 40.9 months were obtained [111]. Using a propensity-matching and a cohort of 308 patients among which only 46 patients received TKI and SBRT, a significant PFS benefit was obtained in comparison with patients treated with TKIs only (*p* = 0.03). No significant OS benefit was shown [108]. These retrospective results were further confirmed in a phase III randomized trial [112]. Among the 631 screened patients, 136 patients were enrolled and randomly assigned to either first-generation TKI (gefitinib, erlotinib or icotinib) alone or upfront RT prior to TKI. With a median follow-up of 23.6 months, 133 patients were analyzable. The majority of patients had one to four metastases (> 85% in both arms). Upfront RT followed by TKI significantly prolonged PFS from 12.5 months (CI 95% 11.6–13.4) to 20.2 months (CI 95% 17.9–22.5) and OS from 17.6 (CI 95% 15.4–19.8) to 25.5 months (23.2–27.8) (*p* < 0.001). A currently ongoing phase II trial (NCT02314364) focuses on the benefit of consolidative SBRT on residual disease in the lung, liver, adrenal glands, and/or spine within 6 months of initiating TKI treatment in patients with oncogene-driven NSCLC (with alterations in EGFR, ALK, ROS1). A phase II study (ATOM) assessed the efficacy of SBRT delivered to residual oligometastases (after 3 months TKI) in 16 patients. When compared to screen-failed patients (unfit for SBRT), patients that benefited from SBRT had a higher PFS (HR 0.41, *p* = 0.01) [113]. This PFS benefit was confirmed by OS in a previously presented multi-institutional phase II trial including 12–20% patients with oncogene-driven NSCLC (41.2 vs. 17.0 months) [14,104].

In case of oligoprogression, RT is considered as a way to overcome treatment resistance and especially resistance to EGFR TKIs. In a phase II trial comparing erlotinib vs. erlotinib + RT in patients experiencing progression after an EGFR TKI [114], RT was associated with a modest PFS of 5.8 months (95% CI 2.5–11.3) and OS of 2.9 years (95% CI 1.1–2.9). The benefit of adding RT to first and second generation TKI must be further explored given the positive results of third generation EGFR TKIs [115]. Several studies in which both PFS and OS benefits were retrospectively [116,117] and prospectively [118] reported, suggesting that local SBRT should be further evaluated in large scale RCTs. SBRT has seen a growing interest for oligoprogressive patients under TKIs [119,120]. Available data remain scarce in this situation [121]. The results of several trials are, however, awaited (NCT01573702; NCT02450591).

### 4.3. Ongoing Trials for Oligometastatic NSCLC Patients

The SARON trial [122] (NCT02417662) is a randomized phase III trial focusing on patients with oligometastatic EGFR, ALK and ROS1 mutation negative NSCLC; the oligometastatic state being defined by the presence of one to three sites of synchronous metastatic disease, among which one must be extracranial. While the control arm is a standard platinum-doublet chemotherapy, the investigational arm will evaluate the benefit of delivering RT to the primary and then the metastatic sites. With 340 awaited patients, the main drawback will be a comparison with a chemotherapy-only based treatment and not a chemo-immunotherapy one. Focusing on a similar clinical setting, the TRAILOCLORI trial (NCT05111197) will evaluate the benefit of stereotactic radiotherapy to oligopersistent sites in NSCLC patients, the disease controlled with long-term immunotherapy. With a more aggressive approach, the CHESS trial (NCT03965468) will evaluate the benefits of a multidisciplinary approach combining PD-L1 inhibitor and chemotherapy as well as SBRT to all metastatic lesions. If there is no disease progression at 3 months, normofractionated radiotherapy will be delivered to the primary tumor while continuing the PD-L1 inhibitor.

With a similar approach but focusing on the primary, the PRIME-LUNG (NCT05222087) will evaluate the benefits of upfront SBRT to the primary in combination with chemo-immunotherapy, compared to chemo-immunotherapy alone as a first-line treatment for de novo stage IV NSCLC patients.

The NIRVANA trial (NCT03774732) is a phase III trial evaluating the benefits of localized radiotherapy (conformational or stereotactic radiotherapy) to the primitive or metastatic lesions in patients treated with a PD-1 inhibitor and concomitant chemotherapy for a stage IV NSCLC.

The LONESTAR trial (NCT03391869) is an ongoing phase III randomized trial evaluating the benefit of local consolidative treatment (LCT) in EGFR/ALK negative NSCLC patients treated with nivolumab + ipilimumab. Of note, the LCT could either be radiotherapy or surgery.

Similar studies are also being conducted in EGFR/ALK/ROS1-mutated patients. In the NORTHSTAR trial (NCT03410043), patients treated with frontline osimertinib are randomized between osimertinib alone vs. osimertinib + consolidation treatment to as many sites as feasible; the primary endpoint being the PFS.

Irrespective of the histology or the mutational status, the SABR-COMET 3 (NCT03862911) and SABR-COMET 10 (NCT03721341) trials are phase III comparing standard of care vs. standard of care + SBRT in patients with, respectively, up to 3 or 10 metastases. These trials and several other trials are further detailed in Table 6 giving an overview of ongoing prospective trials.

Prospective and phase III data supporting the OS benefit of local consolidative radiotherapy in the NSCLC setting remain scarce but tend to favor RT with a low and acceptable toxicity profile. This therapeutic approach remains currently evaluated in several ongoing phase II/III trials and should be offered to patients within clinical trials.

## 5. Conclusions

Despite many retrospective and prospective studies, the local treatment of synchronous metastatic cancer by irradiation of the primary disease for breast or non-small cell lung cancer has not yet been validated as a standard of care. Trials are underway to justify the survival benefit. The challenge will be to determine the group of patients who can benefit from it. In the meantime, the indications must be discussed on a case-by-case basis in a multidisciplinary consultation meeting. In the case of metastatic oligometastatic prostate cancer, the indication for radiotherapy of the primary site has demonstrated a significant increase in overall survival and progression-free survival and is now considered as a standard of care. This indication will be reinforced by ongoing trials. The combination of local treatment of the primary tumor and all metastatic lesions by stereotactic irradiation, particularly in the case of oligometastatic cancer, seems to be an interesting strategy while awaiting the results of the many ongoing trials on this subject.

## Figures and Tables

**Table 1:** Retrospective studies evaluating the impact of local treatment for metastatic breast cancer.

Author	Number of Patients	Local Surgery Number of Patients (%)	Mastectomy Number of Patients or %	Positive Margins	Radiation Therapy	Survival Results with Local Treatment without Local Treatment		*p* Value	Characteristics Associated with Higher OS Rate in Multivariate Analysis
**Khan et al., 2002** [17]	16 023	9162 (57%)	61%	25%	63%	27.7–31.8% (3 years)	17.3% (3 years)	<0.0001	Surgery, systemic treatment, number of metastatic sites
**Rapiti et al., 2006** [18]	300	127 (42%)	72%	11%	89%	27% (5 years specific)	12% (5 years)	0.0002	Age < 60 years, no N3 involvement, ER+, no visceral metastasis, no CNS metastasis, hormonal treatment, surgery with negative margins
**Babiera et al., 2006** [19]	224	82 (37%)	19	31	0	95% (3 years)	79% (3 years)	0.091	Single metastatic site, HER2 +, Caucasian
**Gnerlich et al., 2007** [20]	9734	4578 (47%)	54%	NR	41%	36 months (median)	21 months (median)	<0.001	NR
**Fields et al., 2007** [21]	409	187 (46)	54%	33%	0	26.8 months (median)	12.6 months (median)	0.0005	Surgery, exclusive bone metastatic disease
**Hazard et al., 2008** [22]	111	47 (42.3%)	67%	29%	67%	43% (3 years)	37% (3 years)	NR	NR
**Cady et al., 2008** [23]	622	234 (38%)	NA	NR	NR	44% (3 years)	24% (3 years)	<0.0001	Young patient, HR+, exclusive metastatic bone involvement
**Bafford et al., 2008** [24]	147	61 (41%)	65%	NR	NR	42.2 months (median)	28.3 months (median)	0.093	Surgery, no CNS metastasis, HR+, HER 2+++.
**Blanchard et al., 2008** [25]	395	242 (61%)	77.7%	NR	99.7%	27.1 months (median)	16.8 months (median)	<0.0001	Surgery, ER+, PR+, number of metastatic sites
**Ruiterkamp et al., 2009** [26]	728	288 (39.6%)	6 6%	NR	34%	24.5% (5 years)	13.1% (5 years)	<0.0001	Surgery, age, no more than one metastatic site, no concurrent disease (*p* = 0.06), systemic therapy
**Shien et al., 2009** [27]	344	160 (47)	84%	NR	0	27 months (median)	22 months (median)	0.049	Surgery, age <50 years, soft tissue or bone metastases
**Neuman et al., 2010** [28]	186	69 (37%)	40%	41%	13%	NR	NR	NR	ER+, PR+, HER2+++
**Nguyen et al., 2012** [29]	733	255 (67%)	48.6%	24.3%	RT alone: 22% surgery followed by RT: 11%	21% (5 years)	14% (5 years)	<0.001	Age < 50 years, T1 tumor, RE+, R0 surgery, chemotherapy, hormone therapy, locoregional treatment
**Lang et al., 2013** [30]	208	134 (64.4%)	30.6%	NR	32%	56.1 months (median)	37.2 months (median)	0.002	Chemotherapy
**Thomas et al., 2016** [31]	21372	13042 (61%)	NR	NR	NA	9.6% (10 years)	2.9% (10 years)	<0.001	NR
**Choi et al., 2018** [32]	245	82 (34%)	78%	NR	66%	71% (5 years)	40% (5 years)	<0.001	Endocrine therapy
**Le Scodan et al., 2009** [33]	598	320 (55%)	71 (21%)	49 Gy breast/chest wall Boost 22 Gy	RT alone: 78% surgery followed by RT: 13%	43.4% (3 years)	26.7% (3 years)	0.00002	Single metastatic site, young age, locoregional treatment, no visceral metastases, N0
**Bourgier et al., 2010** [34]	308	239 (80%)	92 (38%)	50 Gy breast/wall with or without boost	RT alone: 62%	RT alone: 39% (3 years) surgery followed by RT: 57% (3 years)	NR	NR	
**Mauro et al., 2016** [35]	125	125	0	50 Gy or hypofractionated 42 Gy: 56% 30 Gy 10 fractions: 40%	RT alone: 100%	23.4 months (median)	NR	NR	Karnofsky, number of metastatic sites, hormone therapy
**Pons-Tostivint et al., 2018** [36]	4276	1706 (40%)	55%	NR	RT alone: 31% surgery followed by RT: 43%	63 months (survivors > 1 year)	43.9 months (survivors > 1 an)	0.006	Locoregional treatment; HR+/HER2-; HER2 +++.

Abbreviations: CI confidence interval, CNS central nervous system, HER2 human epidermal growth factor receptor 2, HR+ hormone receptor positive, ER+ estrogen receptor positive, PR+ progesterone receptor positive, RT: radiation therapy, NR: not relevant, NA: not available.

**Table 2:** Published randomized trials for metastatic breast cancer.

Trial	Number of Patients	Age (Median)	Local Surgery	Mastectomy	Radiation Therapy	Survival Results with Local Treatment without Local Treatment		*p* Value	Locoregional Progression with Local Treatment without Local Treatment		Low Metastatic Burden * OS HR (95%CI)
**Badwe et al., 2015** [43]	350	48 years	173	72%	80%	Median survival: 19.2 months Survival at 2 years: 41.9%	Median survival: 20.5 months Survival at 2 years: 43%.	HR 1.04, (95% CI 0.81–1.34) *p* = 0.79	5.3%	10.6%	1.16 (0.69–1.95)
**Soran et al., 2018** [44]	274	52 years	138	46%	54%	Survival at 3 years: 60% Survival at 5 years 41.6% (46 months)	Survival at 3 years: 51% Survival at 5 years: 24.4% (37 months)	*p* = 0.10 *p* = 0.05	1%	11%	Solitary bone metastases:0.55 (0.36–0.86) Solitary liver/pulmonary metastases: 0.69 (0.37–1.29
**Khan et al., 2022** [45]	256	56 years	125	70%	84%	Survival at 3 years: 68.4% (54.9 months)	Survival at 3 years: 67.9% (53.1 months)	HR 1.11, (90% CI 0.82–1.52); *p* = 0.57	16,3%	39.8%	1.18 (0.38–3.67)

Abbreviations: CI—confidence interval, OS—overall survival. * Low metastatic burden: Badwe et al (Tata Memorial): subgroup of patients with 3 or fewer metastases, Soran et al (MF07-01): oligometastatic subgroups: solitary bone or solitary liver/pulmonary metastasis, Khan et al (E2108): Oligometastasis at registration (exploratory post hoc subgroup analyses).

**Table 3:** Retrospective trials for metastatic prostate cancer.

Author	Number of Patients and Follow Up	Treatment Modalities	Results
**Culp et al., 2014** [51]	*n* = 8185	LT = 374	5 years OS
	Median follow-up: 16 months	RP (*n* = 245)	67.4%
		BT (*n* = 129)	52.6%
		NLT (*n* = 7811)	22.5% (*p* < 0.001)
			RP is associated with CSM in MVA:
			(0.38, CI 0.27–0.53 *p* < 0.001)
**Fossati et al., 2015** [52]	*n* = 8197	LT (*n* = 628) (either RP or RT)	Interaction LT and CSM (*p* < 0.0001)
	Median follow-up: 36 months LT, 31 months NLT	NLT (*n* = 7569)	Reduction in CSM for LT with a predicted 3-year mortality < 40% (*p* < 0.0001)
**Satkunasivam et al., 2015** [56]	*n* = 4069 Median follow-up: 20 months	LT = 242 RP (*n* = 47)	3- year OS 73%
		IMRT (*n* = 88)	72%
		CRT (*n* = 107)	37%
		NLT (*n* = 3827)	34%
			IMRT was associated with a reduction of CSM
			(HR 0.38 CI 0.24–0.61 *p* < 0.001)
**Rusthoven et al., 2016** [53]	*n* = 6382 Median follow-up: 5.1 years	LT = 538 RP (*n* = 69)	5-year OS 49%
		NLT, ADT alone (*n* = 5844)	25%
			*p* < 0.001
**Löppenberg et al., 2016** [54]	*n* = 15501 Median follow-up: 39 months	LT = 1470 RT (*n* = 1131)	3-year OS 60%
		RP (*n* = 294)	78%
		BT (*n* = 45)	80%
		NLT (*n* = 14031)	48%
			*p* < 0.001
			LT was associated with a 39% risk reduction of mortality compared with NLT in MVA adjusted for PSA
			Gleason score, TNM stage, age
**Leyh-Bannurah et al., 2017** [55]	*n* = 13692 Median follow-up: 43.5 months LT, 31 months NLT	LT = 474, NLT = 13218 RT (*n* = 161) RP (*n* = 313)	LT was associated with lower CSM compared with NLT (HR 0.4 IC95% 0.32–0.5)
**Parikh et al., 2017** [57]	*n* = 6051	LT = 827	2-year OS 5-year OS
	Median follow-up: 22 months	RP (*n* = 622)	72.5% 45.7% LT
		IMRT (*n* = 52)	80.6% 17.1% NLT (*p* < 0.01)
		CRT (*n* = 153)	47.6%
		NLT (*n* = 5224)	48.9%
			*p* < 0.0001
**Cho et al., 2016** [58]	*n* = 140	LT = 38	3-year OS
	Median follow-up: 34 months	RT (*n* = 38)	69%
		NLT = 102	43%
			*p* = 0.004

LT: locally treated, NLT: non locally treated, RP: radical prostatectomy, BT: brachytherapy, OS: overall survival, PFS: progression-free survival, RT: radiotherapy, CSM: cancer-specific mortality, IMRT: intensity-modulated radiotherapy, CRT: conformal radiotherapy, MVA: multivariate analysis.

**Table 4:** Ongoing trials for metastatic prostate cancer.

Phase III	Location	Patients Included	Intervention	Outcome	End of Study
PEACE-1 [65]	France	1173	Arm A: ADT + docetaxel Arm B: AA+ADT + docetaxel Arm C: RT+ADT + docetaxel Arm D: AA+RT+ADT + docetaxel	OS PFS	2032
SWOG NCT03678025 [71]	USA	1273	Arm I: Systemic treatment Arm II: Systemic treatment + (RP/RT)	OS	2031
PRESTO [72]	France	350	Arm A: RT + Soc Arm B: Soc	TCR	2027
Phase I I					
PLATON [67]	Canada	410	Arm 1: Systemic treatment + prostate directed therapy if low metastatic burden	PFS	2025
			Arm 2: Systemic treatment+ local treatment of all sites		
LoMPII [68]	Belgium	1273	Arm I: RP+/-ADT	Randomization feasibility	2021
			Arm II: RT+/-ADT		
UHSeste NCT02913859 [69]	Croatia	60	Experimental arm: ADT + LHRHa +/- aA + prostate-pelvic RT	PFS	2020
			Standard arm: ADT alone		
IP2 ATLANTA [70]	UK	918	Arm 1: Systemic treatment	pCR	2024
			Arm 2: Systemic treatment + TAMI	Adverse events	
			Arm 3: Systemic treatment + RP/RT +’metastases	PFS	
MSKCC NCT04262154 [73]	USA	44	Atezolizumab + RT + (aA, prednisone, leuprolide)	2-year FFS	September 2023
MD Anderson NCT01751438 [66]	USA	180	Arm 1: Systemic treatment	PFS	February 2023
			Arm 2: Systemic treatment + RP/RT		

ADT—androgen deprivation therapy, aA—antiandrogen, Soc—standard of care, RP—radical prostatectomy, BT—brachytherapy, OS—overall survival, PFS—progression free survival, RT—radiotherapy, TCR—time to castration resistance (or death from any cause), pCR—complete pathological response.

**Table 5:** Prospective studies of consolidative radiotherapy in metastatic NSCLC patients, irrespective of the mutational status.

Author	Study Type	Number of Patients	Clinical Stage	Percentage of Patients with Targetable Mutations	Modality of RT	Irradiation of the Primitive and/or Metastases	Control Arm	Percentage of Treated Brain Metastases	Follow-Up	Median PFS	Median OS
Gomez et al. [14]	Randomized phase II	49	Synchronous	12–20%	48% SBRT	Primitive and all residual metastatic sites	Maintenance chemotherapy or watching	25%	38.8 months	14.2 vs. 4.4 months (*p* = 0.02)	41.2 vs. 18.9 months (*p* = 0.02)
Palma et al. [106]	Randomized phase II	18/99	Metachronous Controlled primary	Not defined	100% SBRT	All metastatic sites	Standard	2%	51 months	11.6 vs. 5.4 months (*p* = 0.001)	50.0 vs. 28.0 (*p* = 0.006)
Iyengar et al. [107]	Randomized phase II	29	Synchronous	0%	100% SBRT	Primitive and all metastatic sites	No control arm (concomitant chemotherapy)	0%	9.6 months	9.7 vs. 3.5 months (*p* = 0.1)	Not reached vs. 17 months
Bauml et al. [105]	Single arm phase II	45	Synchronous or Metachronous	Not defined	67% SBRT	Primitive and all metastatic sites	No control arm (concomitant pembrolizumab)	36%	25 months	18.7 months	41.6 months
De Ruysscher et al. [95]	Single arm phase II	39	Synchronous	7.7%	0% SBRT to the primitive	Primitive and all metastatic sites	No control arm	43.6%	27.7 months	12.1 months	13.5 months
Collen et al. [96]	Single arm phase II	26	Synchronous or Metachronous	7.7%	100% SBRT	Primitive and all metastatic sites	No control arm	13%	16.4 months	11.2 months	23 months
Petty et al. [97]	Single arm phase II	27	Synchronous	Not defined	Not defined	All sites of residual disease	No control arm	41%	24.2 months	11.2 months	28.4 months
Arrieta et al. [98]	Single arm phase II	37	Synchronous	43.2%	18.9% SBRT	Primitive and all metastatic sites	No control arm	43.2%	32.5 months	23.5 months	Not reached
Wang et al. [108]	Randomized phase II	127	Synchronous	100% mEGFR	100% SBRT	Primitive and all metastatic sites	Standard: first-line TKI	0%	23.6 months	20.2 vs. 12.5 months (*p* < 0.001)	25.5 vs. 17.4 months (*p* < 0.001)

Abbreviations: RT—radiotherapy, PFS—progression-free survival, OS—overall survival, SBRT—stereotactic body radiotherapy, mEGFR—mutated epidermal growth factor receptor, TKI—tyrosine kinase inhibitor.

**Table 6:** Overview of currently ongoing prospective trials in metastatic NSCLC patients, irrespective of the mutational status.

NCT	Clinical Setting	Definition of the Oligometastatic Stage	Study Type	Interventional Arm	Control Arm	Primary Endpoint
NCT03965468	Synchronous oligometastatic and not-mutated NSCLC	≤3 distant metastases One metastasis must be extra-cerebral	Phase II single arm	First phase: PD-L1 inhibitor (durvalumab)Carboplatin + paclitaxelSBRT to all oligometastatic lesions Restaging at 3 months: if no progression, normofractionated to the primary	No control arm	12 months PFS
NCT05278052	Synchronous oligometastatic and not-mutated NSCLC	1–5 metastatic sites ≤3 metastases per organ	Phase III	Standard maintenance therapy + Local RT to all oligometastatic sites including the primary loco-regional disease	Standard maintenance therapy	OS
NCT03391869	Metastatic and not-mutated NSCLC	Not restricted to oligometastatic NSCLC	Phase III	Nivolumab + ipilimumab + local treatment of the primary (surgery of RT) after 2 cycles of immunotherapy	Nivolumab + Ipilimumab	OS
NCT05222087	Metastatic and not-mutated NSCLC	Not restricted to oligometastatic NSCLC	Phase II/III	First-line chemo-immunotherapy +/- SBRT to the primary	Chemo-immunotherapy	OS
NCT02417662	Synchronous oligometastatic and not-mutated NSCLC	1–5 metastatic sites ≤3 metastases per organ	Phase III	Platinum-doublet chemotherapy + RT to the primary and the metastatic sites	Platinum-doublet chemotherapy	OS
NCT03774732	Advanced and not-mutated NSCLC	Not restricted to oligometastatic NSCLC	Phase III	Pembrolizumab + chemotherapy + RT to the primary and the metastatic sites	Pembrolizumab + chemotherapy	OS
NCT04908956	Synchronous oligometastatic and EGFR mutated NSCLC	1–5 metastatic sites	Phase II single arm	Osimertinib + SBRT to the primary tumor and all metastatic sites	No control arm	Safety
NCT05277844	Synchronous oligometastatic and EGFR mutated NSCLC	1–5 metastatic sites ≤3 metastases per organ	Randomized phase II	TKI + SBRT to the primary tumor and all metastatic sites	TKI alone	PFS
NCT03410043	Advanced and EGFR mutated NSCLC	Not restricted to oligometastatic NSCLC	Randomized phase II	Osimertinib + Local treatment (Surgery or RT) to the primary and/or metastatic sites	Osimertinib	PFS
NCT03705403	Oligometastatic NSCLC	1–5 metastatic sites ≤2 brain metastases	Randomized phase II	RT to all metastatic sites + immunocytokine L19-IL2 (darleukin)	Standard of care: systemic treatment or local treatment (RT or surgery) or wait and see	PFS
NCT05111197	Oligopersistent and not-mutated EGFR	1–5 metastatic sites ≤3 brain metastases	Randomized phase III	PD-1 or PD-L1 inhibitor + SBRT to metastatic and persistent sites	PD-1 or PD-L1 inhibitor	OS
NCT03827577	Oligopersistent or oligorecurrent with controlled primary	1–3 metastatic sites	Randomized phase III	Standard medical treatment + LAT (SBRT, RFA or surgery)	Standard medical treatment	OS
NCT03862911	Oligopersistent or oligorecurrent with controlled primary	1–3 metastatic sites	Randomized phase III	Standard medical treatment + SBRT to metastatic and persistent sites	Standard medical treatment	OS
NCT03721341	Oligopersistent or oligorecurrent with controlled primary	4–10 metastatic sites	Randomized phase III	Standard medical treatment + SBRT to metastatic and persistent sites	Standard medical treatment	OS
NCT03137771	Metastatic NSCLC stable under standard medical treatment	Not restricted to oligometastatic NSCLC	Randomized phase II/III	Maintenance therapy + SBRT/RT to a single extracranial site	Maintenance therapy	Phase II: PFS Phase III: OS
NCT03256981	Oligoprogressive mutated EGFR	1–3 oligoprogressive sites	Randomized II	Continued TKI therapy + SBRT	Continued TKI therapy	PFS
NCT04405401	Oligoprogression on ICI or TKI	1–5 metastatic sites	Randomized II	Continued therapy + SBRT	Standard medical treatment	PFS/OS
NCT02756793	Oligoprogressive NSCLC	1–5 metastatic sites	Randomized II	Continued therapy + SBRT	Standard medical treatment	PFS

Abbreviations: NCT—National Clinical Trial number, NSCLC—non-small cell lung cancer, PD-L1—programmed death ligand 1, SBRT—stereotactic body radiotherapy, PFS—progression-free survival, RT—radiotherapy, OS—overall survival, TKI—tyrosine kinase inhibitor, LAT—local ablative therapy, RFA—radiofrequency ablation, ICI—immune checkpoint inhibitor.
